# Outer Membrane Vesicles of *Helicobacter pylori *TK1402 are Involved in Biofilm Formation

**DOI:** 10.1186/1471-2180-9-197

**Published:** 2009-09-15

**Authors:** Hideo Yonezawa, Takako Osaki, Satoshi Kurata, Minoru Fukuda, Hayato Kawakami, Kuniyasu Ochiai, Tomoko Hanawa, Shigeru Kamiya

**Affiliations:** 1 Department of Infectious Disease, Kyorin University School of Medicine, Shinkawa, Mitaka, Tokyo, 181-8611, Japan; 2 Laboratory for Electron Microscopy, Kyorin University School of Medicine, Shinkawa, Mitaka, Tokyo, 181-8611, Japan; 3 Department of Anatomy, Kyorin University School of Medicine, Shinkawa, Mitaka, Tokyo, 181-8611, Japan; 4 Department of Bacteriology, Nihon University School of Dentistry, Kanda-Surugadai, Chiyoda-ku, Tokyo, 101-8310, Japan

## Abstract

**Background:**

*Helicobacter pylori *forms biofilms on glass surfaces at the air-liquid interface in *in vitro *batch cultures; however, biofilms of *H. pylori *have not been well characterized. In the present study, we analyzed the ability of *H. pylori *strains to form biofilms and characterized the underlying mechanisms of *H. pylori *biofilm formation.

**Results:**

Strain TK1402 showed strong biofilm forming ability relative to the other strains in Brucella broth supplemented with 7% FCS. The strong biofilm forming ability of TK1402 is reflected the relative thickness of the biofilms. In addition, outer membrane vesicles (OMV) were detected within the matrix of only the TK1402 biofilms. Biofilm formation was strongly correlated with the production of OMV in this strain. We further observed that strain TK1402 did not form thick biofilms in Brucella broth supplemented with 0.2% β-cyclodextrin. However, the addition of the OMV-fraction collected from TK1402 could enhance biofilm formation.

**Conclusion:**

The results suggested that OMV produced from TK1402 play an important role in biofilm formation in strain TK1402.

## Background

*Helicobacter pylori *is a spiral, microaerophilic, noninvasive, gram-negative bacterium that colonizes the human gastrointestinal tract, primarily the stomach [1]. This organism has been identified as an aetiological agent of chronic active gastritis, peptic ulcer disease [2,3], gastric adenocarcinoma [4], and mucosa-associated lymphoid tissue (MALT) lymphoma [5]. A number of factors such as the VacA cytotoxin, the *cag *pathogenicity island (*cag *PAI), motility, and the urease enzyme are known to be involved in the virulence of this organism [6-8].

Biofilm development is initiated when bacteria transit from a planktonic state to a lifestyle in which the microorganisms are firmly attached to biotic or abiotic surfaces, and biofilms are strongly implicated in bacterial virulence [9]. Biofilm formation is critical not only for environmental survival but also for successful infection by numerous pathogenic bacteria. Among human bacterial pathogens, the biofilms of *Pseudomonas aeruginosa*, *Haemophilus influenzae*, pathogenic *Escherichia coli*, *Vibrio cholerae*, staphylococci and streptococci are some of the best studied [10-14]. Bacterial biofilms are frequently embedded in a self-produced extracellular matrix [15]. The extracellular polymeric substance (EPS) matrix, which can constitute up to 90% of the biofilm biomass, is a complex mixture of exopolysaccharides, proteins, DNA and other macromolecules [16].

Previous studies have alluded to the ability of *H. pylori *to form biofilms [17,18]. A polysaccharide-containing biofilm has been observed at the air-liquid interface when *H. pylori *was grown in a glass fermenter [17]. *H. pylori *is also capable of binding to a heterotrophic mixed species biofilm grown on stainless steel coupons [18]. Recent studies indicated that 10 strains including some animal-adapted strains, clinical isolates and laboratory strains, were able to form similar three-dimensional architectures implicated in biofilm development [19,20]. Cellini *et al*. reported that an environmental *H. pylori *strain, named MDC1, displayed a well structured biofilm [19]. Cole *et al*. also indicated that mucin greatly accelerated planktonic growth relative to the expansion of *H. pylori *biofilms [2]. In addition, a recent study indicated that *H. pylori *can exist in human gastric mucosa forming biofilms [21]. These studies indicated that the topic of biofilm formation in this organism has the potential to contribute to our knowledge of *H. pylori *pathogenesis. However, little is known regarding the mechanism of *H. pylori *biofilm development. In the present study, we characterized the ability of 4 reference strains and 4 clinical isolates of *H. pylori *to form biofilms. Furthermore, we investigated the potential role of outer membrane vesicles (OMV) released from this organism in biofilm development.

## Results

### Biofilm formation by *H. pylori *strains

**Figure 1:**
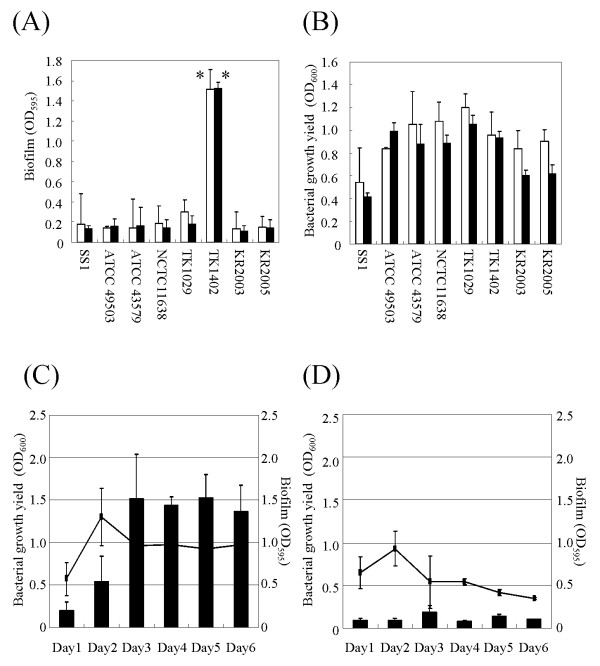
**(A) Biofilm formation by eight *H. pylori *strains**. The graph shows quantification of biofilms formed after 3-days (white bars) and 5-days (black bars) following culture in Brucella broth containing 7% FCS. (B) Eight *H. pylori *strains were grown in Brucella broth containing 7% FCS-, and OD_600 _absorbance was measured at 3-days (white bars) and 5-days (black bars). (C, and D) Time course experiments for the *H. pylori strains *TK1402 (C) and SS1 (D) biofilm formation and their growth. The biofilm mass of these strains are shown as black bars and the lines depict the OD_600 _absorbances of these strains. All of the results were expressed as the means ± standard deviations from at least three independent experiments. *significantly different (*p *< 0.05) relative level of biofilm formation (strain TK1402 versus other strains).

We attempted to grow biofilms of the 8 strains of *H. pylori *on glass coverslip surfaces in Brucella broth supplemented with 7% FCS with shaking for 3 days or 5 days and found that all strains formed biofilms at the liquid-gas interface of the cultures. Under these conditions, all of the strains except strain TK1402 formed relatively little biofilm biomass (Fig. 1A). In contrast, the clinically isolated strain TK1402 showed significantly higher levels of biofilm formation (Fig. 1A). The growth yields of these strains for 3- or 5-days of culturing were comparable for all of the strains (Fig. 1B). To determine the kinetics of *H. pylori *biofilm formation, strains TK1402 and SS1 were assessed for biofilm forming ability and growth rates from day 1 to day 6 (Fig. 1C and 1D). Both strains showed similar growth kinetics with both strains fully grown within 2 days although the maximum titers of strain SS1 were slightly lower compared to that of strain TK1402. After 3 days of incubation, the growth yields were slightly decreased and plateaued at day 6. On the other hand, biofilm formation by strain TK1402 increased until day 3 (Fig. 1C). After 3 days of incubation, biofilm formation reached a plateau up to day 6. Biofilm formation by strain SS1 was not significantly different from day 1 to day 3 (Fig. 1D), and biofilm formation was significantly lower than that of TK1402 upon cultivation for up to 6 days.

### Morphological analysis of biofilms

**Figure 2:**
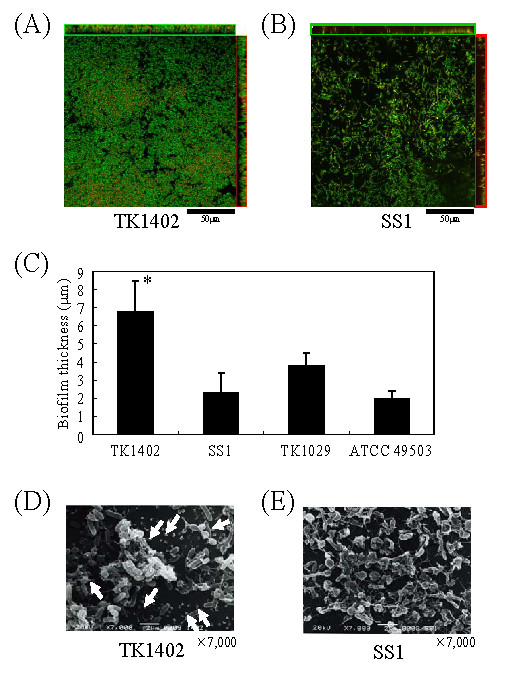
**CLSM images of *H. pylori *strains TK1402 (A) and SS1 (B) biofilms in Brucella broth containing 7% FCS**. Each image represents the layer in the Z-stack that has the maximum bacterial coverage. The 3-day biofilms of each strain were stained with BacLight LIVE/DEAD stain. Viable cells are colored green and nonviable cells are colored red. *x-z *and *y-z *reconstructions of each biofilm are shown on the right and upper sides of each *x-y *image. The scale bars equal 50 μm in both panels. (C) Biofilm thickness of each strain. Each biofilm was scanned with CLSM at five randomly selected positions and *x-z *color detection, corresponding to biofilm thickness, was determined throughout the height of the biofilm. Data are representative of three independent experiments. The results are expressed as the means ± standard deviations. SEM images of *H. pylori *strains TK1402 (D) and SS1 (E) biofilms in Brucella broth containing 7% FCS. The 3-day biofilm of each strain on cover glass was investigated using SEM. The OMV-like structures are indicated by white arrows (D). Scale bars (2 μm) are shown at the bottom of each electron micrograph. *significantly different relative levels of biofilm thickness (*p *< 0.05; strain TK1402 versus other strains).

The biofilms were stained with a BacLight LIVE/DEAD bacterial viability kit solution which could differentiate between live cells (green) and dead cells (red). Strain TK1402 formed strong biofilms covering the entire visible area (Fig. 2A) but the other strain SS1 formed relatively poor biofilms (Fig. 2B). In the biofilms of both strains, the majority of the biofilm cells were stained green (Fig. 2A, and 2B). In order to confirm that the TK1402 biofilm cells were viable, the 2-day and 3-day biofilms cells were scrapped into PBS and the optical densities and the CFU values of the mixtures were evaluated (Table 1). The 2-day and 3-day cultures of this strain in Brucella broth supplemented with 7% FCS were also measured as controls. The optical densities and CFU of the 3-day biofilm cells showed increases compared to those of 2-day biofilm cells. Further, the CFU values were normalized to optical densities. The values for the 2-day biofilm cells were similar compared to the controls (broth culture). However, the normalized values for 3-day biofilm cells tended to be decreased, although there was no significant difference in the normalized values, suggesting that 3-day biofilm cells might contain some dead cells or morphologically altered cells.

**Table 1:** Optical densities and CFU measurements in the strain TK1402 biofilm cells and broth cultures

	OD_600_	CFU (×10^9^)	CFU/OD_600 _(×10^9)^
2-Day biofilm^a^	0.141 ± 0.037	0.259 ± 0.202	1.860 ± 1.487
3-Day biofilm^a^	0.258 ± 0.027	0.340 ± 0.230	1.614 ± 0.695
2-Day broth culture^b^	0.939 ± 0.012	1.847 ± 0.318	1.966 ± 0.387
3-Day broth culture^b^	1.075 ± 0.044	2.248 ± 1.170	2.151 ± 1.180

^a) ^the biofilm cells were scrapped by mechanical treatment into PBS. The cell suspensions were examined by optical densities and CFU were also calculated.

^b) ^the standard broth cultures of strain TK1402 in Brucella broth supplemented with 7% FCS were examined by optical densities and CFU.

Data are shown as the mean value of the 3-independent experiments ± standard deviations.

Next we analyzed the biofilm thickness of strains TK1402, SS1, TK1029, and ATCC 49503 with CMLS observations. Strain TK1402 exhibited 2-fold or greater biofilm thickness compared to the other strains (Fig. 2C). To clarify the architectural characteristics of *H. pylori *biofilms, we compared TK1402 and SS1 biofilms by SEM analysis. In the biofilms of strain SS1, the bacteria attached to glass surfaces in thin layers (Fig. 2E). Interestingly, the biofilms consisted mainly of bleb-like or amorphous structures. On the other hand, the TK1402 biofilms were composed primarily of cells with bacillary morphology which were clearly outlined (Fig. 2D). In addition, these later bacteria showed layer formation with bacterial aggregates in the biofilms. The biofilm bacterial aggregates appeared to result from direct cell-cell attachment. Intriguingly, TK1402 biofilms showed the presence of many OMV-like structures on the glass surface as well as on the bacterial cell surfaces (Fig. 2D, white arrows). These structures were not detected in the biofilms of the other strains (Fig. 2E and data not shown). A recent report indicated that OMV production from *H. pylori *clinical isolate MDC1 was apparent under SEM observation [19]. We thus decided to focus our attention more on the OMV-like structures in subsequent experiments.

### Potential role of the OMV in TK1402 biofilm formation

**Figure 3:**
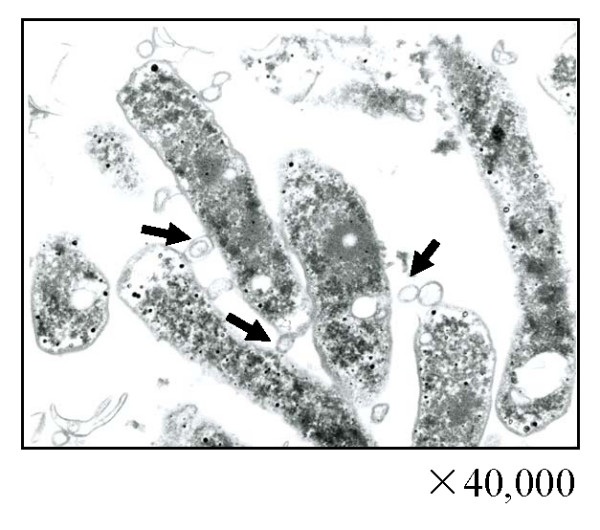
**TEM images of *H. pylori *strain TK1402 biofilms in Brucella broth supplemented with 7% FCS**. The 3-day biofilm of strain TK1402 on glass slides was investigated by using TEM.

We observed more closely the OMV-like structures in the thin-sectioned biofilms using TEM (Fig. 3). These structures consisted primarily of bilayered proteolipids which were mainly spherical in shape (Fig. 3, black arrows). These structures also exhibited the characteristics typical of Gram negative bacterial OMV [22]. We confirmed that the OMV-fraction did not contain flagella by observation with SM and Western blotting with anti-flagella antibody.

**Figure 4:**
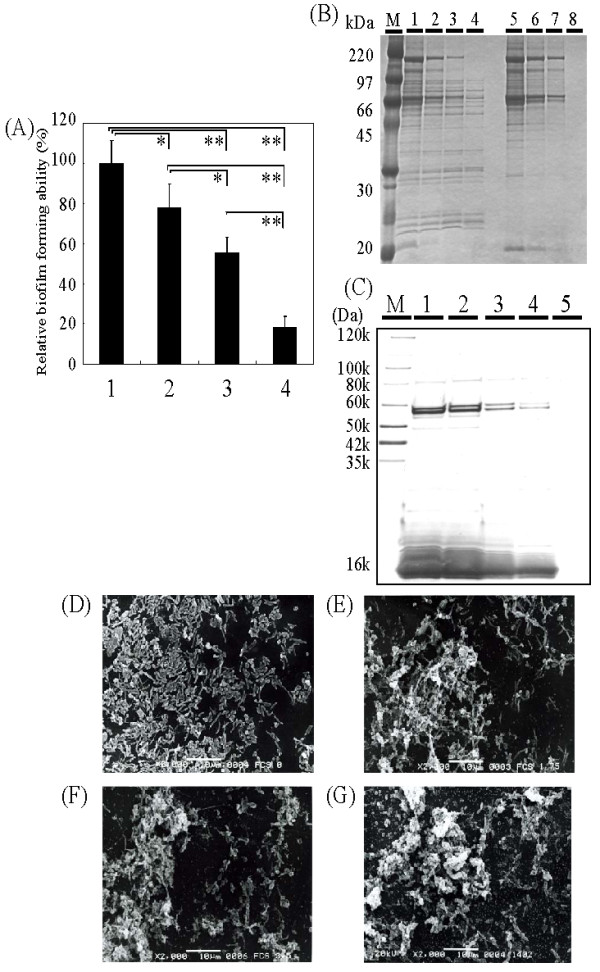
**(A) Effects of FCS concentrations in the biofilm growth medium on TK1402 biofilm formation**. Strain TK1402 biofilms in Brucella broth supplemented with various concentrations of FCS (7%: lane 1, 3.5%: lane 2, 1.75%: lane 3 and 0: lane 4) were examined. Quantification of biofilms (percent) was calculated relative to that of strain TK1402 in Brucella broth supplemented with 7% FCS, which was set equal to 100%. The values for the biofilms under these conditions are shown as in Fig. 1A. (B) The OMV were fractionated from different medium conditions for TK1402 cultures and the OMV-fractions were separated by SDS-PAGE (lane 1, 7% FCS; lane 2, 3.5%; lane 3, 1.75% lane 4, Brucella broth only) and compared to controls (medium without the organism, FCS concentrations were 7%: lane 5, 3.5%: lane 6, 1.75%: lane 7 and 0: lane 8). (C) Western blotting of OMV-fraction from different medium conditions using anti-*H. pylori *antibody. M: Molecular weight marker. Lanes: 1, 7% FCS; 2, 3.5%; 3, 1.75%; 4, 0; 5, 7% FCS without organism (negative control). (D to G) SEM observation of TK1402 biofilms under different medium conditions. D: Brucella broth only (without FCS, 0); E: with 1.75% FCS; F: with 3.5% FCS; G: with 7% FCS. *significantly different (*p *< 0.05). ** significantly different (*p *< 0.005).

We next found that the FCS concentration in the biofilm growing medium affected biofilm formation of *H. pylori *TK1402 (Fig. 4A). The lower concentrations of FCS (3.5%, 1.75% or 0) caused a significant decrease in biofilm formation in this strain compared to that in 7% FCS. Therefore, the intensity of biofilm formation was dependent upon the concentration of FCS. The OMV were isolated from the cells under these conditions and characterized by SDS-PAGE (Fig. 4B). As the components of FCS might be present in the OMV fraction, the control fractions from Brucella broth supplemented with various concentration of FCS (7%, 3.5% 1.75% and 0) without the microorganism were used as controls. There were many protein bands which did not conform to FCS components (Fig. 4, lanes 1 to 4 vs. lanes 5 to 8). To quantify the production of OMV under these conditions, the OMV-fractions were analyzed by Western blotting with anti-*H. pylori *strain NCTC 11638 antibody. There were many positive bands and the intensity of these bands correlated with the FCS concentrations (Fig. 4C). As a negative control, control fractions from Brucella broth supplemented with 7% FCS without the microorganism were used and there were no detectable corresponding bands (Fig. 4C, lane 5). In addition, we observed the biofilms under these conditions with SEM (Fig. 4D to 4G). There were no OMV in the biofilms of Brucella medium only (Fig. 4D). In contrast, a large number of the OMV were detected in biofilms in Brucella broth supplemented with 7% FCS (Fig. 4G). Under these conditions, the quantity of the OMV in the biofilm appeared to be dependent upon the concentration of FCS (Fig. 4D to 4G). These results suggested that the production of OMV might be related to the biofilm forming ability of strain TK1402.

**Figure 5:**
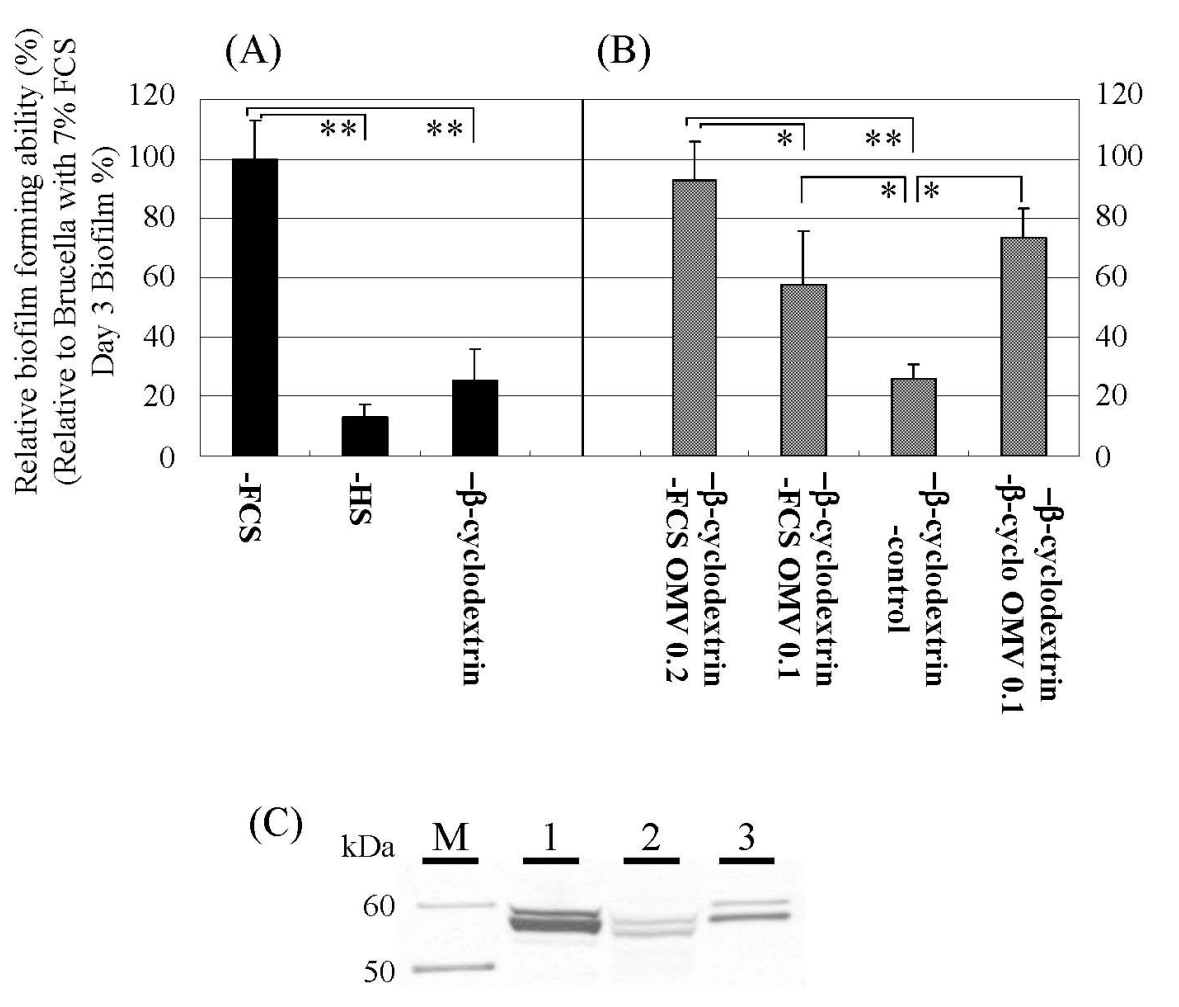
**(A) Biofilm formation by strain TK1402 in Brucella broth supplemented with 7% FCS (-FCS), 7% HS (-HS), or with 0.2% β-cyclodextrin (-β-cyclodextrin)**. Relative biofilm forming activity (percent) was calculated relative to the 3-day biofilm in Brucella broth supplemented with 7% FCS. Data are expressed as the means of all of experiments ± standard deviations. (B) The OMV-fraction was added to Brucella broth supplemented with β-cyclodextrin. The protein concentrations in the OMV-fractions were adjusted and 0.2 mg of the OMV-fraction (β-cyclodextrin-FCS OMV 0.2), or 0.1 mg of the OMV-fraction (β-cyclodextrin-FCS OMV 0.1) were added. Control fractions from the medium without bacteria were also added (β-cyclodextrin-control). Further, the OMV-fraction was isolated from this organism in Brucella broth supplemented with 0.2% β-cyclodextrin and 0.1 mg of the OMV-fraction from 0.2% β-cyclodextrin medium was added (β-cyclodextrin-β-cyclo OMV 0.1). Biofilm formation was examined after 3 days of culture. Relative biofilm forming activity (percent) was calculated relative to the 3-day biofilm in Brucella broth supplemented with 7% FCS. Data are expressed as the means of all of experiments ± standard deviations. (C) Western blotting of the OMV-fraction from different medium conditions using anti-*H. pylori *antibody. M: Molecular weight marker. Lanes: 1, 7% FCS; 2, 7% HS; 3, 0.2% β-cyclodextrin. *significantly different (*p *< 0.05). ** significantly different (*p *< 0.005).

We further determined that 3-day biofilm formation with strain TK1402 in Brucella broth supplemented with 7% HS or 0.2% β-cyclodextrin was significantly weaker than that in Brucella broth supplemented with 7% FCS (Fig. 5A). Other strains, which form thin biofilms in Brucella broth supplemented with 7% FCS, also formed weaker biofilms, similar to or weaker than those in FCS broth with either horse serum or β-cyclodextrin. The final densities of strain TK1402 evaluated by OD_600 _units after 3 days of culture were 0.96 ± 0.09, 1.11 ± 0.19, and 0.87 ± 0.13 following growth with Brucella broth supplemented with 7% FCS, 7% HS, or 0.2% β-cyclodextrin, respectively. We then isolated the OMV from TK1402 cultured in Brucella broth containing 7% FCS, 7% HS, or 0.2% β-cyclodextrin and Western blotting with the anti-*H. pylori *antibody was carried out (Fig. 5C). The 50- to 60-kDa OMV protein band intensities from growth in Brucella broth supplemented with 7% FCS were much greater than comparable fractions from 7% HS or 0.2% β-cyclodextrin-grown cultures. These results suggested that lower production of OMV might lead to weaker biofilm formation in Brucella broth supplemented with 7% HS or 0.2% β-cyclodextrin.

To directly verify that the OMV were components of the TK1402 biofilm matrix and that the production of the OMV can induce strong biofilm formation, TK1402 biofilm formation with 0.2% β-cyclodextrin medium was analyzed following the addition of the OMV fraction from TK1402 cultures in Brucella broth containing 7% FCS. The protein concentration of the OMV-fraction was adjusted to 2.0 mg/ml or 1.0 mg/ml. The OMV fraction (total amounts were 0.2 mg or 0.1 mg, respectively) was added to Brucella broth with 0.2% β-cyclodextrin and biofilm formation with strain TK1402 was carried out. As the components of FCS might be present in the OMV fraction and could affect biofilm formation, a control fraction from Brucella broth supplemented with 7% FCS without the microorganism was used. The levels of biofilm formation in the 0.2% β-cyclodextrin medium supplemented with the control OMV fraction was similar to that of the 0.2% β-cyclodextrin medium alone (Fig. 5B, lane β-cyclodextrin-control). On the other hand, the addition of the 0.1 mg OMV fraction from TK1402 showed significantly higher levels of biofilm formation than those in 0.2% β-cyclodextrin medium with the control fraction (Fig. 4B, β-cyclodextrin-FCS OMV 0.1). The levels of biofilm formation with OMV addition were similar to that in Brucella broth supplemented with 7% FCS (Fig. 4B. β-cyclodextrin-FCS OMV 0.2). We further determined that the 0.1 mg OMV fraction from *H. pylori *cultured in Brucella broth containing 0.2% β-cyclodextrin could also enhance biofilm formation but at levels lower than 0.2 mg of this fraction. The OMV fraction induced more biofilm formation than 0.1 mg of the OMV fraction from 7% FCS medium (Fig. 5B, β-cyclodextrin-β-cyclo OMV 0.1).

### Evaluation of biofilm formation by other isolated *H. pylori *strains

**Figure 6:**
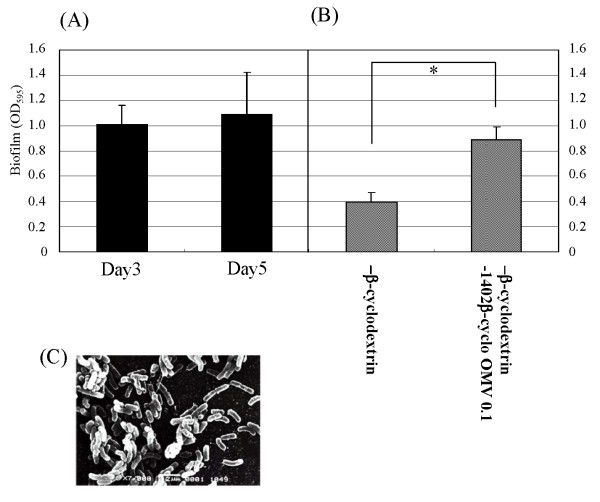
**(A) Biofilm formation by strain TK1049**. Graph shows quantification of biofilms formed after 3-day (Day 3) and 5-days (Day 5) in Brucella broth supplemented with 7% FCS. (B) Biofilm formation by strain TK1049 in Brucella broth supplemented with 0.2% β-cyclodextrin and addition of the OMV-fraction from TK1402 grown in 0.2% β-cyclodextrin medium. (C) SEM observation of TK1049 biofilms. *significantly different (*p *< 0.05).

In order to detect other strains having similar biofilm forming ability to strain TK1402, we assessed the biofilm forming ability of ten additional clinical isolates of *H. pylori*. Only strain TK1049 showed similar levels of biofilm formation to that of strain TK1402 (Fig. 6A). The other strains showed lower levels of biofilm formation than strain TK1402 (the biofilm OD_595 _values ranged from 0.1 to 0.3). The structure of TK1049 biofilms was then observed by using SEM (Fig. 6C). Cellular aggregation was observed to be similar to that of TK1402 biofilms and many vesicle-like structures were also detected with TK1409. Moreover, 3-day biofilm formation with strain TK1049 in Brucella broth supplemented with 0.2% β-cyclodextrin was weaker than that in Brucella broth supplemented with 7% FCS. However, the addition of the OMV fraction from TK1402 in Brucella broth supplemented with 0.2% β-cyclodextrin restored biofilm formation similar to that in Brucella broth supplemented with 7% FCS (Fig. 6B).

## Discussion

In this study, we characterized biofilm formation in *H. pylori *strains and demonstrated differential abilities to form biofilms in reference and clinical isolates. In addition, the production of OMV in strain TK1402 was correlated with strong biofilm formation. Several reports indicated that *H. pylori *has the ability to form biofilms on abiotic surfaces in vitro as well as on human gastric mucosa [18-21,23]. The results of the biofilm formation analyses demonstrated that strain TK1402 has strong biofilm forming ability compared to other strains independent of its growth rate. Development of strain TK1402 and SS1 biofilms from day 1 to day 6 demonstrated that it took 3 days for biofilm maturation under these conditions, suggesting that *H. pylori *biofilm formation might proceed in an organized fashion through early (Day 1), intermediate (Day 2) and maturation (after Day 3) phases of development. Similar distinct developmental phases have been reported for biofilm formation by other bacterial species [24,25]. Since development of biofilms is closely associated with the generation of a matrix, the majority of which is extracellular material, biofilm development in *H. pylori *appears to share common basic steps with other biofilm forming bacteria.

The biofilm forming cells at day 3 generally appeared to be viable when the cells were exposed to Live/Dead BacLight staining. In addition, the normalized CFU values for the biofilm and broth culture cells following 2 days of incubation were comparable. In 3-day biofilm cells, this value was slightly decreased compared to 3-day broth culture cells, suggesting the presence of some dead cells in the biofilm. These results are consistent with the maturation phase of the development of biofilms in 3-day biofilms of strain TK1402, since biofilms are thought to be encased in an EPS matrix as well as dead cells [26]. In addition, strain TK1402 exhibited thick biofilm formation. The biofilm morphology of strain TK1402 showed direct cell-cell bound aggregates as well as flagella-dependent binding forms. The cell-cell interacting forms might act as precursors for thick biofilm formation. Gots *et al*. indicated that cell-cell aggregation induces a multilayered architecture during *Staphylococcus epidermidis *biofilm formation [27]. Moreover, in our SEM observations, for the majority of the *H. pylori *strains examined, *ie*., SS1, biofilms may contain autolysed cells. On the other hand, there were clearly intact cells in TK1402, as well as TK1049, biofilms and the later is also another strong biofilm forming strain. These observations suggested that these strong biofilm forming strains may remain in an active metabolic state for a relatively long time without exhibiting morphological changes or autolysis, in comparison with the other strains. These later properties could be responsible for the weaker biofilm forming activities of most of the strains examined in this study.

In the SEM observations of TK1402 biofilms, there were many OMV. OMV production is a physiologically normal function of gram-negative bacteria [22,28]. It was also reported that the *H. pylori *strains released OMV into the extracellular space [29,30]. A recent report indicated that OMV were a component of the matrix of *P. aeruginosa *biofilms [14]. We analyzed thin-sections of strain TK1402 biofilms with TEM. The OMV were located at the substratum-bacterium interface and extracellular space. Interestingly, some of OMV appeared to be involved in attaching one cell with another. This observation suggested that the OMV produced by strain TK1402 could be intimately involved in biofilm formation.

Previously, several reports indicated that VacA, urease and lipopolysaccharides are present on the surface of OMV from *H. pylori *along with other outer membrane proteins [29,30]. We quantified OMV production in Brucella broth supplemented with various concentration of FCS using Western blotting with anti-*H. pylori *antibody. Moreover, the SEM observations were also carried out to directly confirm this. The FCS concentration in the biofilm medium showed a direct positive correlation with OMV production as well as biofilm forming ability. Further, similar results were detected by the addition of serum from different hosts as well as with synthetic substrates. On the other hand, observation with biofilm forming bacteria indicated that LPS plays a role in biofilm development and architecture [14,31]. Recently, Keenan *et al*. reported that LPS detected in OMV under iron-limited conditions were notably shorter than those under iron-replete conditions [32]. We hypothesize that strain TK1402 has an altered LPS, particularly LPS O-antigens under different experimental conditions including the use of different animal sera, synthetic substrates, or different FCS concentrations. To confirm this, we analyzed the LPS profiles of *H. pylori *cultured in different culture media by SDS-PAGE and silver staining. However, there were no differences in the LPS O-antigen profiles. We then isolated the OMV from the TK1402 culture supernatant in order to examine the role of these structures in biofilm formation. Biofilm formation by this strain was increased following the addition of the OMV-fraction in a dose-dependent manner. Although the quantities of OMV added were three- to five-fold more than the quantity of the OMV which exist in biofilms under our experimental conditions, the OMV appear to play an important role in the formation of the extracellular matrix of strain TK1402 biofilms. The extracellular matrix serves a role in bacterial attachment to abiotic and cellular surfaces in the initial stage of biofilm formation [33]. It is possible that specific proteins in the OMV released from strain TK1402 may take part in bacterial aggregation and biofilm formation. Which component(s) of the OMV contribute to biofilm formation still remains to be determined. Additional investigations are now in progress to determine such components in the OMV.

In the present study, we searched for other clinical isolates with strong biofilm formation and one strain, TK1049, exhibited similar ability to form biofilms as strain TK1402. This suggested that *H. pylori *strains exhibiting strong biofilm forming ability are relatively rare. Indeed, Williams *et al*. indicated that FCS inhibited adherence to abiotic surfaces in some of the *H. pylori *strains [34]. This apparent discrepancy between their study and our present results in terms of the effects of FCS might be due to differences in the *H. pylori *strains used.

Strain TK1402 was isolated from a patient with duodenal and gastric ulcers in Japan. This strain contains the *cagA*, *cag*PAI and *vacA *genes as demonstrated by PCR [35]. It was also shown that this strain expresses the Lewis^y ^antigen (LeY) on the cell surface. Moreover, strain TK1402 was reported to exhibit virulence in gnotobiotic mice [36], C57BL mice [37], and Mongolian gerbils [35]. These reports indicated that the TK1402 strain has the ability to colonize the stomach of these animals as well as in humans. These results as well as our present findings suggest that this colonization ability might be correlated with the strong biofilm forming ability of strain TK1402. Therefore, we speculate that strong biofilm forming ability is related to gastric colonization by *H. pylori *in various animals as well as in humans. It is recognized that an understanding of *H. pylori *biofilm formation is still in its infancy. The ability of *H. pylori *strains, as exemplified by strain TK1402, to form biofilms may play a part of role in the infectious process.

## Conclusion

We have demonstrated that strain TK1402 has strong biofilm forming ability. In addition, the results suggested that this property is dependent upon direct cell-cell binding mediated by the OMV of this strain. This represents a new observation relative to a potentially novel gastric cell colonization factor of this organism.

## Methods

### Bacterial strains and culture conditions

The following *H. pylori *strains were used: SS1, ATCC 49503, ATCC 43579, NCTC11638, TK1029, TK1402, KR2003, and KR2005. The last four are clinical isolates from Japanese patients. Strains TK1029 and TK1402 were used as described previously [38]. In addition, strains TK1036, TK1042, TK1043, TK1045, TK1046, TK1047, TK1049, TK1054, TK1056, and TK1057 were also used for assessing biofilm forming ability. Strains KR2003 and KR2005, as well as the latter strains were isolated from a gastritis patient in our laboratory. All strains were maintained at -80°C in Brucella broth (Difco, Detroit, Mich) with 20% (vol/vol) glycerol. These strains were cultured under microaerobic conditions at 37°C on Brucella agar plates containing 7% horse serum (HS).

### Biofilm formation and its quantification

Biofilm formation by all strains was carried out as previously described [19,20] with slight modifications. Briefly, sterilized glass coverslips (approximately 22 × 22-mm, 0.12 to 0.17-mm thickness, Matsunami Glass, Tokyo, Japan) were placed into 12-well microtiter plates. Each well was filled with 2 ml of Brucella broth supplemented with 7% fetal calf serum (FCS), 7% horse serum (HS), or 0.2% β-cyclodextrin to allow adherence of *H. pylori *at the air-liquid interface. The formation of biofilms was initiated by inoculating 10 μl of pre-cultured cell suspension (approximately 5 × 10^5 ^cells in Brucella broth) into each well. The cultures were incubated under microaerobic conditions at 37°C for 1 to 6 days with shaking (80-100 rpm). After incubation, the coverslips were removed and washed with phosphate-buffered saline (PBS). The samples were then air dried and stained with crystal violet for 30 s. After being stained, the coverslips were rinsed with distilled H_2_O to remove excess dye and then air dried for 30 min. All dye associated with the biofilms was dissolved with 1 ml of ethanol and 200 μl of the ethanol solutions were used to measure the absorbance at 594 nm with a microplate reader to determine the amount of biofilm formation.

### Confocal laser scanning microscopy (CLSM) and measurement of biofilm thickness

For visualization, the biofilms of *H. pylori *strains on the coverslips were stained with a BacLight LIVE/DEAD bacterial viability kit solution (Molecular Probes, Leiden, The Netherlands) according to the directions of the supplier. Confocal images were collected by using a Zeiss LSM 510-META confocal laser scanning microscope (Carl Zeiss, Jena, Germany). To determine biofilm thickness, a series of horizontal (*xz*) optical sections at 0.5 μm intervals were taken through the height of the biofilm for measurement. Each biofilm was scanned at five randomly selected positions. Each sample was observed independently more than three times. Confocal images of green and red fluorescence were constructed simultaneously using a multitrack mode.

### Cell viability assay

To determine the numbers of viable bacteria, biofilm cells on the coverslips were scrapped into PBS. The optical densities and colony-forming units (CFU) of the cell suspensions were quantitated as the mean of three independent observations. As controls, standard cell broth cultures were used.

### Electron microscopic studies

To observe the biofilm ultrastructure, the biofilms formed on the coverslips were examined by scanning electron microscopy (SEM). The biofilms on the coverslips were fixed with 2% glutaraldehyde for 3 h at room temperature and the samples were observed using a JSM-5600LV electron microscope (JEOL, Tokyo, Japan).

To observe the OMV-like structures, the biofilms of strain TK1402 on the glass slides were examined by using transmission electron microscopy (TEM). Glass slides cut in half were placed into 6-well microtiter plates and the biofilms were allowed to form as described above. The biofilms were fixed with 2% glutaraldehyde for 3 h at room temperature. The samples were then dehydrated and embedded in Epon 813 embedding solution (Chemische Werke Lowi GmbH, Waldkaraigurg, Germany). The sections were finally observed with a JEM-100 electron microscope (Jeol).

### Isolation of outer membrane vesicles

Isolation of OMV was performed as described previously [30]. Briefly, *H. pylori *strain TK1402 was grown in Brucella broth supplemented with various concentration of FCS for 3 days. After cultivation, the optical density at 600 nm of the cell cultures was adjusted to 0.5 with each respective medium. The cells were collected by centrifugation (10,000 g for 15 min), and the resulting supernatants were filtered (low protein binding Durapore membrane, 0.45 mm polyvinylidene fluoride, Millipore, Bedford, Mass.). The filtrates were centrifuged (40,000 g, 2 h at 4°C), washed with PBS and re-centrifuged (40,000 g, 2 h at 4°C). The pellets were next resuspended in PBS supplemented with 0.2 M NaCl. The media without the bacteria were used as controls. The OMV of strain TK1402 in Brucella broth supplemented with 0.2% β-cyclodextrin or 7% horse serum were also isolated in a similar manner.

### Sodium dodecyl sulfate-polyacrylamide gel electrophoresis (SDS-PAGE) and immunoblotting techniques

The fractionated OMV (OMV-fraction) were treated with sodium dodecyl sulfate (SDS) loading buffer including 5% 2-mercaptoethanol at 100°C for 5 min and separated by polyacrylamide gel electrophoresis (PAGE). The separated OMV proteins were stained with Coomassie brilliant blue. For Western blotting assays, the OMV-fractions were loaded onto gels and transferred to polyvinylidene difluoride membranes (Atto, Tokyo, Japan). After transfer, the membranes were blocked with 3% bovine serum albumin in PBS for 60 min and incubated with *H. pylori *strain NCTC 11638 whole-cell antiserum (1:2,000) [36] for 60 min. After washing with PBS containing 0.05% Tween 20 (PBST), peroxidase-labeled goat anti-rabbit immunogloblins (Dako A/S, Glostrup, Denmark) were used at 1:2,000 dilution as secondary antibodies. After washing with PBST, the blots were developed.

### Complementation of biofilm forming ability using the OMV

The OMV-fraction from Brucella broth supplemented with 7% FCS (OMV-fraction) and the medium fraction (control-fraction) in PBS were adjusted to an optical density of 2.0, or 1.0 at 280 nm. The OMV-fractions from Brucella broth supplemented with 0.2% β-cyclodextrin were also adjusted to optical densities of 1.0. After filtration, 100 μl of the fractionated OMV were added to Brucella broth with 0.2% β-cyclodextrin for TK1402 biofilm formation assays (described above).

### Statistical analysis

Statistical analysis was performed using the Mann-Whitney U test. P values of 0.05 or less were considered to indicate statistical significance.

## Authors' contributions

HY carried out the experiments and drafted the manuscript. TO contributed to the experimental concept and design as well as provision of technical support. SKu initially conceived the idea for this study. MF carried out the microscopy techniques while HK and KO participated in discussions regarding the study design. TH contributed to the experimental concept and design as well as assisting in technical support. SKa was also involved in the conception of this study, and participated in its design and coordination as well as helping to draft the manuscript. All authors read and approved the final manuscript.
